# Morpho-molecular identification and first report of *Fusarium equiseti* in causing chilli wilt from Kashmir (Northern Himalayas)

**DOI:** 10.1038/s41598-021-82854-5

**Published:** 2021-02-11

**Authors:** Ammarah Hami, Rovidha S. Rasool, Nisar A. Khan, Sheikh Mansoor, Mudasir A. Mir, Nazeer Ahmed, Khalid Z. Masoodi

**Affiliations:** 1 Transcriptomics Laboratory (K-Lab), Division of Plant Biotechnology, Sher-e-Kashmir University of Agricultural Sciences and Technology of Kashmir, Shalimar, Srinagar, Jammu and Kashmir 190025 India; 2 Division of Plant Pathology, Sher-e-Kashmir University of Agricultural Sciences and Technology of Kashmir, Shalimar, Srinagar, Jammu and Kashmir 190025 India

**Keywords:** Biotechnology, Plant sciences

## Abstract

Chilli (*Capsicum annuum* L.) is one of the most significant vegetable and spice crop. Wilt caused by Fusarium Sp. has emerged as a serious problem in chilli production. Internal transcribed spacer (ITS) region is widely used as a DNA barcoding marker to characterize the diversity and composition of Fusarium communities. ITS regions are heavily used in both molecular methods and ecological studies of fungi, because of its high degree of interspecific variability, conserved primer sites and multiple copy nature in the genome. In the present study we focused on morphological and molecular characterization of pathogen causing chilli wilt. Chilli plants were collected from four districts of Kashmir valley of Himalayan region. Pathogens were isolated from infected root and stem of the plants. Isolated pathogens were subjected to DNA extraction and PCR amplification. The amplified product was sequenced and three different wilt causing fungal isolates were obtained which are reported in the current investigation. In addition to *Fusarium oxysporum* and *Fusarium solani*, a new fungal species was found in association with the chilli wilt in Kashmir valley viz., *Fusarium equiseti* that has never been reported before from this region. The studies were confirmed by pathogenicity test and re-confirmation by DNA barcoding.

## Introduction

Chilli (*Capsicum annuum* L.) is considered as the most important vegetable and spice crop throughout the world which belongs to Genus Capsicum, and family “Solanaceae”^1^. Chillies have gained their commercial importance in the horticulture industry because of their color and pungency. These are rich source of proteins and vitamins mainly Vitamin A, C and E. Chillies also possess important health benefits due to their antioxidant and anti-inflammatory properties because of the presence of various bioactive molecules like volatile oils, fatty acids, carotenoids and capcaicinoids^2–4^. Chilli is cultivated throughout the world over an area of 1832 thousand hectares producing 2959 thousand tons^5^. The prime chilli producing countries are China, India, Pakistan, Korea, Indonesia, Turkey and Sri Lanka in Asia; Egypt, Tunisia, Ghana and Nigeria in Africa; Mexico, United States of America in North—Central America; Spain, Yugoslavia, Romania, Italy, Bulgaria and Hungary in Europe and Peru and Argentina in South America^6^. Among all chilli grown countries, India is the largest chilli producing country followed by China and Pakistan^7^. The largest producer of Chilli in the world is India with 1400 thousand tonnes of production^8^, followed by China with 450 thousand tonnes and Mexico with 400 thousand tonnes^9,10^.

However, the chilli production has been suffering to a great extend because of many bacterial, viral and fungal diseases. More than 40 diseases of chillies are known to be caused by fungi^11^. One of the important diseases is Chilli wilt to which major loss can be attributed. The common species causing chilli wilt are *Phytopthora, verticulum wilt, Rizoctonia root rot and Fusarium wilt*^12,13^. As per the reports *Fusarium oxysporum* and *Fusarium solani* are considered the most common fungal species are found associated with Chilli wilt in India^14,15^ along with *Fusarium moniliforme* and *Fusarium palidoroseum* which are reported to be found in some parts of the country^16^. Total yield loss due to *Fusarium* wilt has been estimated to be from 10 to 80% in total chilli production on a global level on the basis of selected varieties and climatic conditions^17^. The disease caused by *Fusarium oxysporum* and *Fusarium solani* is a serious complication that reduces growth, fruit yield and quality threatening chilli production^18^. The common symptoms of fusarium wilt are discoloration and inward rolling of the leaves followed by the wilting of the whole plant which is caused due to the damage in vascular system thus hampering nutrient and water uptake^19–21^. *Fusarium equiseti* is considered to be a weak pathogen on cereals and is occasionally found to be associated with *Fusarium* head blight infected kernels^22^.This species is commonly found in tropical and sub-tropical areas^23^. The species is a pathogen to varied range of crops and it has been recently reported to be a causal organism of wilt in *Capsicum chinense* in Mexico^24^. The pathogenicity of this species is has underestimated though. The species belongs to the *F.incarnatum-F. quiseti* complex and it is genetically diverse. However, various investigations have been carried out on chilli wilt causing pathogens and several management strategies has been designed for controlling the chilli wilt.

*Fusarium* species have several morphological characteristics that help these for distinct identification and one of the prominent features is development of various shapes and sizes of macro and micro conidia which are asexual spores. Other structures that they form are called chlamydospores spores^25^ which ensure the survival of the pathogen in soil and in plant for many years thus making the management and control of the diseases caused by *Fusarium* species very difficult to cater^26,27^. These are also identified on the basis of growth rate on agar media and the pigmentations produced by them^28^. Moreover, the morphological identification can be quite difficult among the *Fusarium* species^29^. Identification of the species is done through macro and microscopic analysis but the most reliable form of identification is through the information gathered via nucleotide sequencing from conserved gene regions which include Internal Transcribed Spacer (ITS)^30^. The sequence information using ITS regions has been immensely used in phylogeny and taxonomy of *Fusarium* species^31^ as ITS regions have been known to successfully aid in identification between the species^32^. ITS is differentiated into two regions ITS1 and ITS2 (genes 18S to 5.8S and 5.8S to 28S respectively)^33^. There are more than 172,000 fungal ITS sequences present in Genbank^34^.

A deep comprehension of the populations of pathogens is important as they show variations in pathogenicity, response to management systems, environment and host differences^35^. Thus population biology of the pathogen needs to be studied with depth. Since, the incidence of wilt is very prevalent in Kashmir so it is the need of an hour to do a detailed investigation of pathogen causing chilli wilt. It has not been yet cleared that among the population of *Fusarium* species how many are responsible for causing chilli wilt in Kashmir region. In this regard major chilli growing hotspots, pathogens association and prevalence of major pathogen in different areas of Kashmir Himalayas were investigated and morphological and molecular studies were carried out which, for the first time revealed *Fusarium equiseti* to be one of the causal organisms for chilli wilt in Kashmir along with *Fusarium oxysporum, Fusarium Solani.*

## Materials and methods

### Collection, Purification and Maintenance of culture

Survey of vegetable growing areas in four districts of Kashmir valley viz., Srinagar, Budgam, Pulwama and Anantnag was carried out to assess the prevalence of the disease (Table 1).The fungi from the infected samples were isolated using tissue bit technique^36^. The isolated pathogens were purified using hyphal tip and / or single spore technique^37^. Pure cultures thus obtained were maintained by repeated sub- culturing at intervals of 45 days for further studies and the sterile cultures were stored at 4 °C in a refrigerator.

**Table 1:** Accession number of isolates in NCBI Genbank collected from different places.

Sr. no	Coding of isolates	Place of collection	Pathogen identified	Accession numbers
1	A1	Budgam (Narakara)	*Fusarium equiseti*	MK503775.1
2	A2	Budgam (Soibugh)	*Fusarium equiseti*	MK503776.1
3	A3	Pulwama (Muran)	*Fusarium equiseti*	MK503777.1
4	A4	Pulwama (Parigam)	*Fusarium equiseti*	MK503778.1
5	A5	Pulwama (Kangan)	*Fusarium solani*	MK503834.1
6	A6	Srinagar (Shalimar)	*Fusarium solani*	MK503835.1
7	A7	Anantnag (Achabal)	*Fusarium solani*	MK503836.1
8	A10	Anantnag (Bulbulnowgam)	*Fusarium oxysporum*	MK503838
9	A11	Anantnag (Brakpora)	*Fusarium oxysporum*	MK503840
10	A12	Budgam (Sabdan)	*Fusarium equiseti*	MK503841.1

### Identification and Pathogenicity test

Identification of the pathogen was confirmed on the basis of morphological and the pathological characteristics. Identification was confirmed from division of Plant Pathology, SKUAST-K and also confirmed through DNA barcoding. The pathogen inoculum was multiplied on sand meal agar medium. The medium was prepared by autoclaving 90 g dry sieved sand and 10 g maize meal with 40 ml of distilled water at 1.05 kg cm-2 pressure for half an hour for three consecutive days. The sterilized medium was then inoculated with respective fungi and incubated for three weeks at 25 ± 1 °C with daily shaking of flasks to get uniform growth. The inoculums thus prepared were added to the sterilized sand soil (2:1) potting mixture @ 10 per cent (w/w) by mixing it with upper layer of soil and allowed for 7 days to infest soil^38^ (Raj and Singh 1973; Najar 2001). Forty five days old chilli seedlings (cv. ‘Shalimar Long’), raised by growing surface sterilized seeds in sterilized sand soil (2:1) potting mixture were gently uprooted and transplanted in the sterilized potting mixture incorporated in sterilized soil served as check. The study on symptom development of chilli wilt disease was undertaken on artificially inoculated potted chilli plants (cv. ‘Shalimar Long’). The chilli seedlings transplanted in infested soil, inoculated with the test pathogen were continuously monitored for symptom development till the plants were completely dead.

### Morphological and cultural characteristics of the isolated pathogen(s)

The morphological characteristics of the causal pathogen(s) were studied *in-vivo* after culturing on artificial medium to identify the associated pathogen(s). The pathogen cultures were grown on potato dextrose agar (PDA) medium and the semi- permanent slides prepared from 10 days old colonies. The important characters studied were:

- Hyphae, width, septation and colour
- Conidia, shape, size and colour
- Conidiophore, shape, size and colour
- Chlamydospore, shape, size and colour.

### DNA Extraction and PCR Amplification

**Figure 1:**
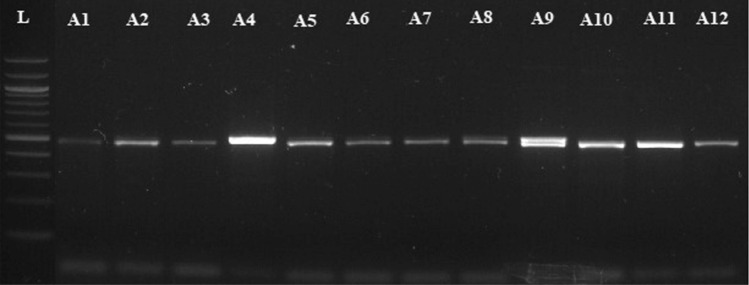
PCR product amplified from ITS Region in *Fusarium* sp. L-100 bp ladder, all samples have shown significant amplification of expected band size.

DNA extraction was carried out using 400 microlitre extraction buffer. This buffer is same as that described by Cenis et al.^39^. The genomic DNA of fungal isolates was loaded on 0.7% agarose in 1XTAE for 30 min. PCR amplification was carried out using ITS1 andITS2 primer pair for the amplification of all the three isolates. The primers were designed manually using, Oilgocalc, Clustalw software the primer pairs used for amplification were ITS1F Primer (5′CCTGCGGAGGATCATTA 3′), ITS2R Primer (5′TCCTCCGCTTATTGAT3′). PCR amplification for ITS1 5.8S-ITS2 region was carried out in a reaction volume of 25 µl in 0.2 ml PCR tubes. The amplification reaction was carried out in thermo cycler (*Applied Biosystems*) for 1- 2 h for amplification of ITS1 -ITS2 region followed by gel electrophoresis procedure and compared with 100 bp DNA ladder, respectively (Fig. 1).

### Sequencing, Nucleotide Alignment and Phylogenetic Analysis

Amplified PCR products were sequenced at Agri Genome Labs (Infopark Road, Kakkanad, Kerala, India). The same primers utilized for the PCR amplification were used for sequencing as well. PCR products (sequences) were assembled using the DNA Baser V.4 program to produce complete contigs. These were further aligned using the CLUSTAL W method (Bio-Edit). A search of homologous sequences was performed by BLAST analysis at NCBI (http://ncbi.nlm.nih.gov/BLAST). The MEGA7 (Molecular Evolutionary Genomics Analysis Version 7)^40^ constructed dendrograms from the 10 isolates from the current study and reference strain sequences from GenBank. There were 100 replications for every bootstrap value. For validation of results, an out group fungal pathogen *Alternaria* was selected.

## Results

Diseased plant samples were collected from four districts of Kashmir region of India viz., Srinagar, Budgam, Pulwama and Anantnag on the basis of symptomatology, during 2018–2019. The characteristic symptoms discovered were lesions on the root/stem, brown tube-shaped structure discoloration, yellowing, weakening and death of the plants.

### Morphological characterization and identification of the *Fusarium solani, Fusarium oxysporum* and *Fusarium equiseti*

Morphological characteristics of the causal pathogens were studied both on host as well as on artificial culture medium to identify the associated pathogen which were studied on PDA medium.

**Figure 2:**
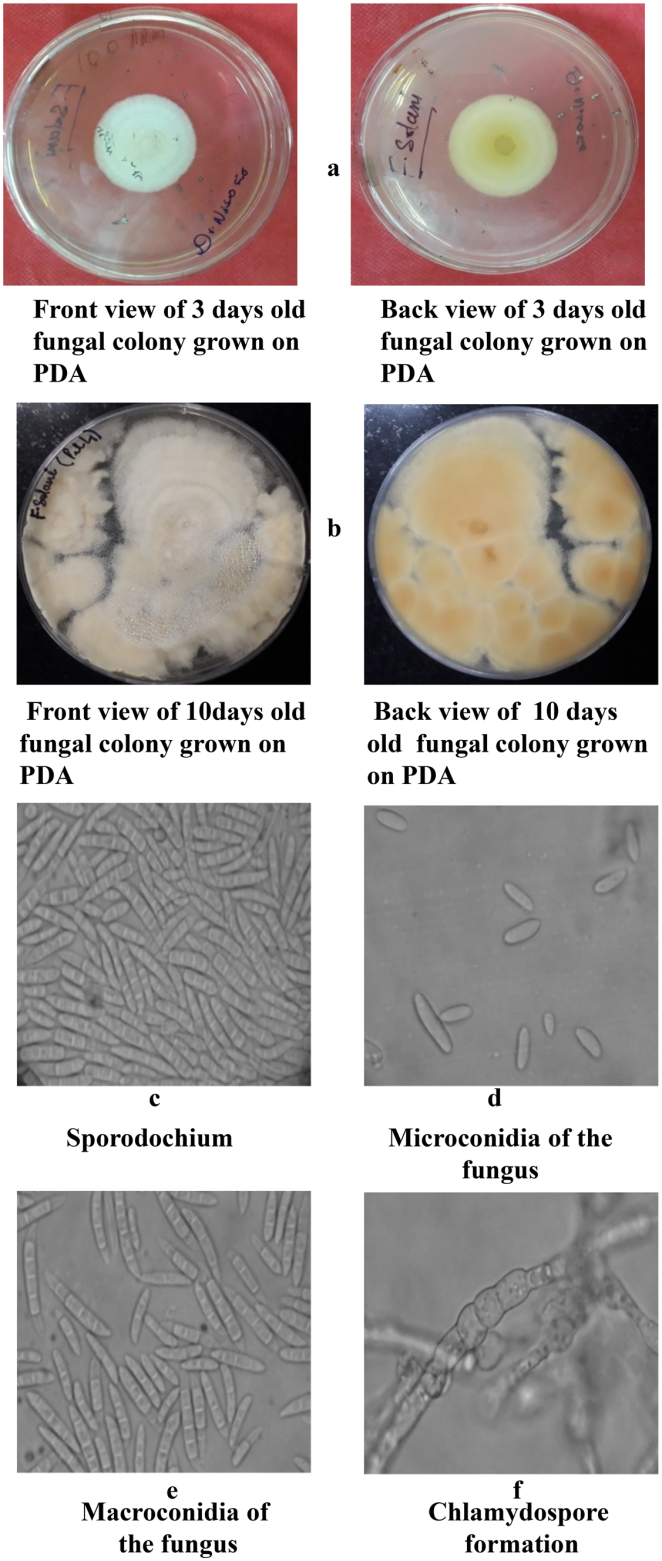
Morpho—cultural characteristics of *Fusarium solani* (Mart.) Sacc. Causing chilli wilt.

**Figure 3:**
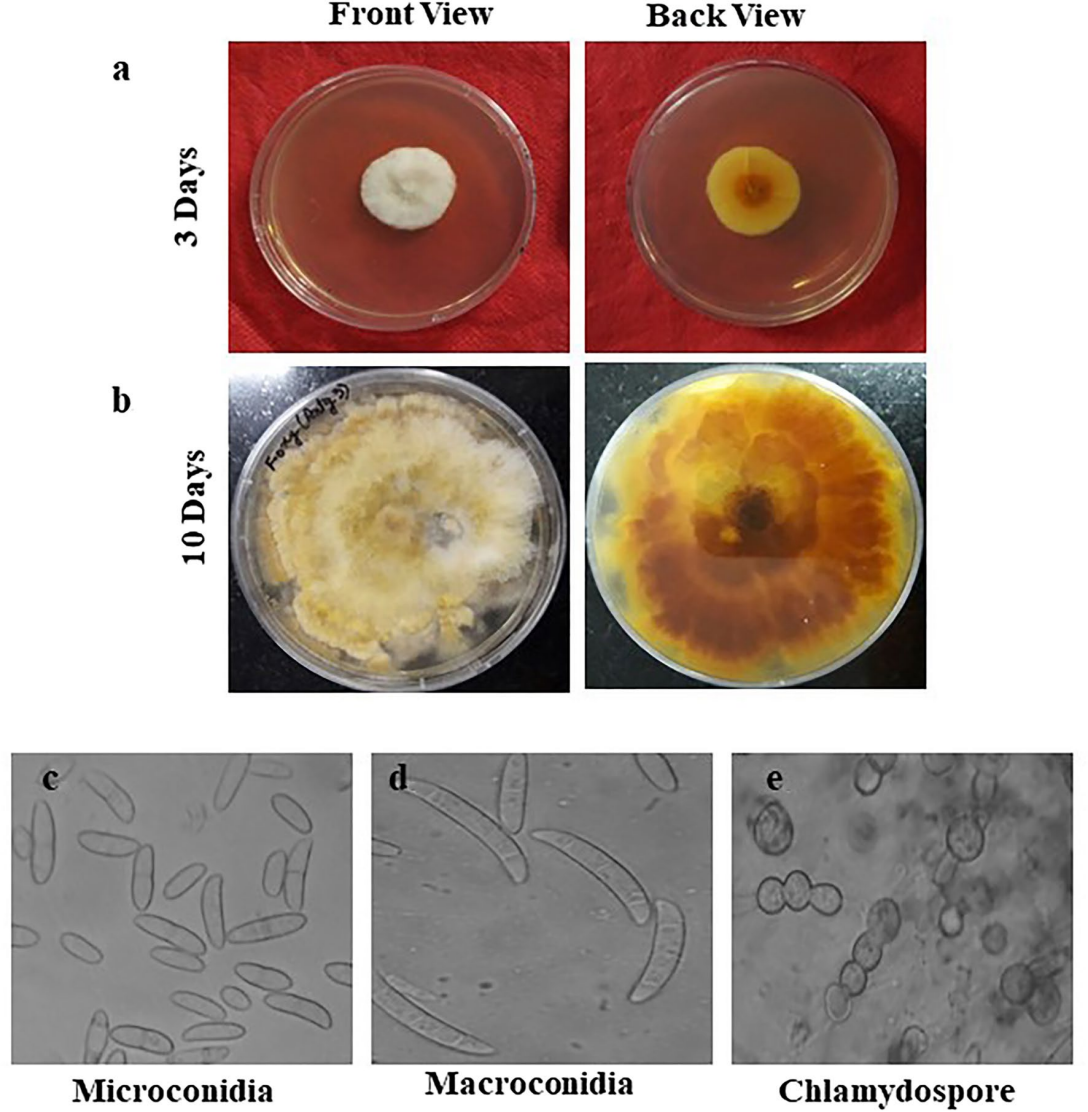
Morpho—cultural characteristics of *Fusarium oxysporum* Schlecht. Causing chilli wilt.

**Figure 4:**
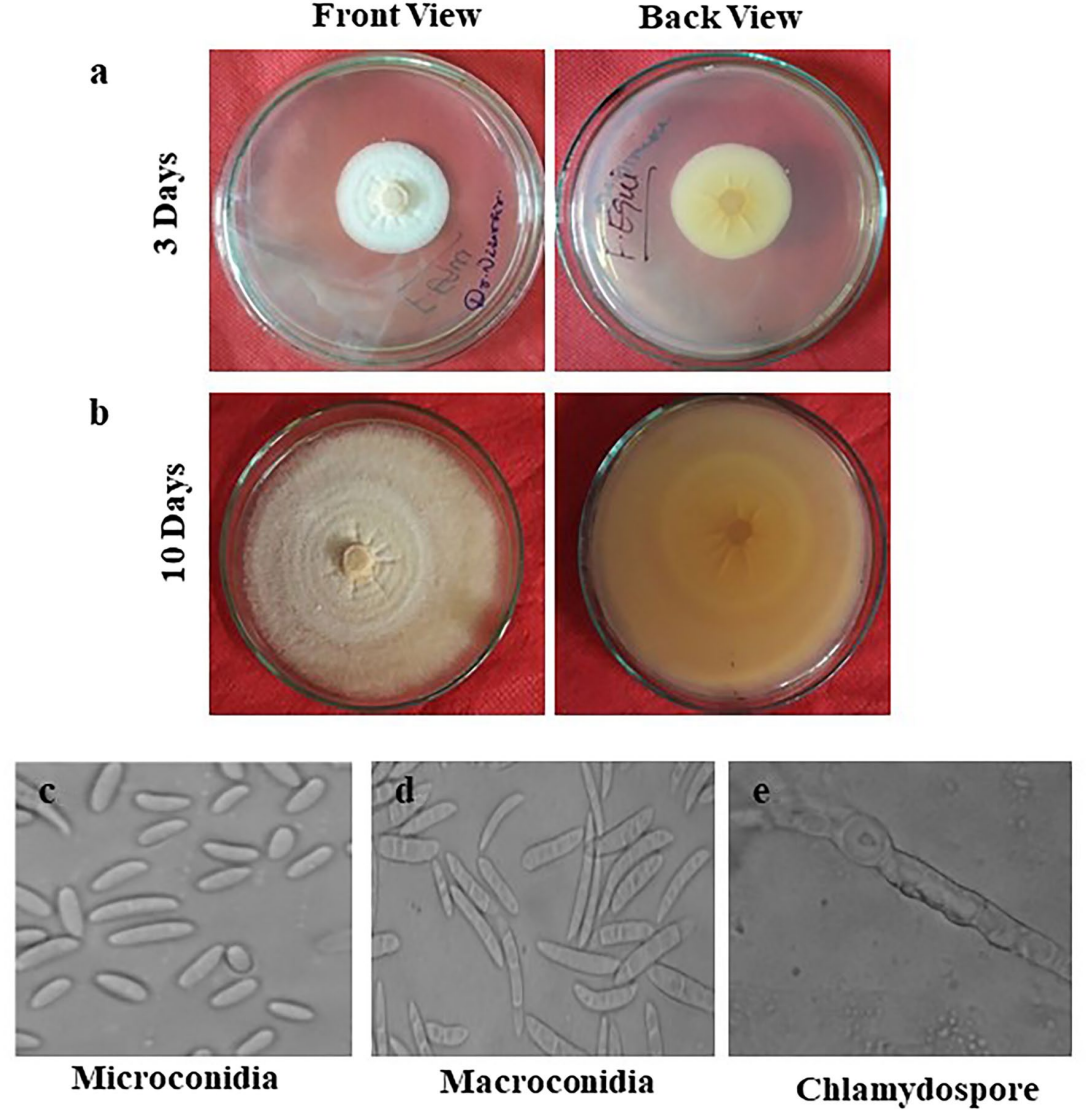
Morpho—cultural characteristics of *Fusarium equiseti* (Corda.) Causing chilli wilt.

The pure culture of the *Fusarium solani* initially produced white colonies (Fig. 2a) which gradually turned to light orange coloured at agar base (Fig. 2b) and attained a growth of 90 mm in 10 days of incubation at 25 ± 1 °C. Microscopic observations revealed that mycelium was smooth, branched, cylindrical, septate and 3.50- 5.50 µm in width. Conidiophores were cylindrical, short, simple, septate and measured 88.60–110.50 × 2.50–4.50 µm in size. Microconidia were round to oval in shape, hyaline, 0–1 septate, measuring 7.50–11.00 × 2.80–3.75 µm in size (Fig. 2c) Macroconidia were curved with short apices pedicellate basal cells, hyaline, 3–4 septate, measuring 21.15–32.00 × 3.80–4.75 µm in size (Fig. 2d,e). Clamydospores were intercalary, produced singly or in chains, nearly spherical, hyaline and measured 5.80–9.00 µm in diameter (Fig. 2f). Like *Fusarium solani,* pure cultures of *Fusarium oxysporum* produced white colonies at the beginning (Fig. 3a) which later turned to peach brown colour at agar base (Fig. 3b) and attained a growth of 90 mm in 10 days of incubation at 25 ± 1 °C. Mycelium was recorded to be smooth, cylindrical, septate and branched with 3.00- 4.80 µm in width. Conidiophores were measured to be 87.40–112.50 × 2.50–5.00 µm in size. Microconidia were ellipsoidal to cylindrical, straight or curved in shape, born on short philaids, hyaline, 0–1 septate and measuring 6.80–16.00 × 3.50–4.00 µm in size (Fig. 3c). Macroconidia were fusiform, pointed at ends, pedicellate basal cells, hyaline, 2–4 septate and measuring 32.50–44.00 × 4.00–5.50 µm in size (Fig. 3d,e). Clamydospores were produced terminally, singly or in chains, nearly spherical, hyaline and measured 5.00–11.00 µm in diameter (Fig. 3f). in the case of *Fusarium equiseti* pure culture produced white colonies (Fig. 4a) later turning to peach orange colour at agar base (Fig. 4b) with a growth of 90 mm in 10 days of incubation at 25 ± 1 °C. Microscopic observations revealed that mycelium was smooth, branched, cylindrical, septate and 3.25- 4.80 µm in width. Conidiophores were cylindrical, short, simple, septate and measured 72.50–106.30 × 3.00–4.5.0 µm in size. Microconidia were oval, hyaline, 0–1 septate and measuring 12.00–16.00 × 3.20–4.50 µm in size (Fig. 4c). Macroconidia were curved with tapered and elongated apical cell, prominent foot cell, hyaline, 2–5 septate and measuring 24.00–40.00 × 4.00–6.00 µm in size (Fig. 4d,e). Clamydospores were produced intercalary, singly or in chains, nearly spherical, hyaline and measured 5.00- 11.00 µm in diameter. Morphological characters of *Fusarium solani, Fusarium oxysporum* and *Fusarium equiseti* are represented in Tables 2, 3 and 4 respectively.

**Table 2:** Morpho-cultural characteristics of *Fusarium solani* (Mart.) Sacc. Causing chilli wilt.

Fungal propagule	Shape	Colour	Size	Septation
Colony	Floccose, Scant aerial mycelium	Initially white, turning light orange coloured at agar base	90 mm dia. In 10 days	-
Hyphae	Smooth, cylendrical and branched	Hyaline	3.50–5.20 µm in width	Septate
Conidiophores	Cylendrical, short and simple	Hyaline	88.60–110.50 × 2.50- 4.50 µm	Septate
Microconidia	Round to oval	Hyaline	7.50–11.00 × 2.80–3.75 µm Av. (9.75 × 3.20 µm)	0-1Septate
Macroconidia	Curved with short , pedicellate basal cells	Hyaline	21.15–32.00 × 3.80–4.75 µm Av. (28.50 × 4.30 µm)	3- 4 Septate
Clamydospores	Spherical, smooth and rough walled, Produced terminally, intercalary, single or in chains	Hyaline	5.80–9.00 µm in dia	Aseptate

**Table 3:** Morpho-cultural characteristics of *Fusarium oxysporum* Schlecht. Causing chilli wilt.

Fungal propagule	Shape	Colour	Size	Septation
Colony	Floccose, abundant aerial mycelium	Initially white, turning felted, peach brown at agar base	90 mm dia. In 10 days	-
Hyphae	Smooth, cylindrical and branched	Hyaline	3.00—4.80	Septate
Conidiophores	Cylindrical, short and simple	Hyaline	87.40–112.50 × 2.50- 5.00 µm	Septate
Microconidia	Ellipsoidal to cylindrical, straight or curved borne on short simple philaids	Hyaline	6.80–16.00 × 3.50–4.00 µm	0-1Septate
Macroconidia	Fusiform, pointed at both ends, basal cells pediculate	Hyaline	32.50–44.00 × 4.00–5.50 µm	2- 4 Septate
Clamydospores	Spherical, smooth and rough walled, Produced terminally, latterly or intercalary	Hyaline	5.00–11.00 µm	-

**Table 4:** Morpho-cultural characteristics of *Fusarium equiseti* (Corda.) Sacc. Causing chilli wilt.

Fungal propagule	Shape	Colour	Size	Septation
Colony	Floccose, abundant aerial mycelium	Initially white, turning peach orange at agar base	90 mm dia. In 10 days	-
Hyphae	Smooth, cylindrical and branched	Hyaline	3.25—4.80 µm in width	Septate
Conidiophores	Cylindrical, short and simple	Hyaline	72.50–106.30 × 3.00- 4.50 µm	Septate
Microconidia	Oval	Hyaline	12–16 × 3.20– 4.50 16.00 µm Av. (13.00 × 3.85 µm)	0-1Septate
Macroconidia	Curved with tapered and elongated apical cell; prominent foot cell	Hyaline	24.00–40.00 × 4.00–6.00 µm Av. (3.00 × 5.30 µm)	2- 5 Septate
Clamydospores	Spherical, smooth and rough walled, Produced terminally, latterly or intercalary or in pairs, frequently forming chains	Hyaline	5.00–11.00 µm	-

### Pathogenicity and Symptomatology

**Figure 5:**
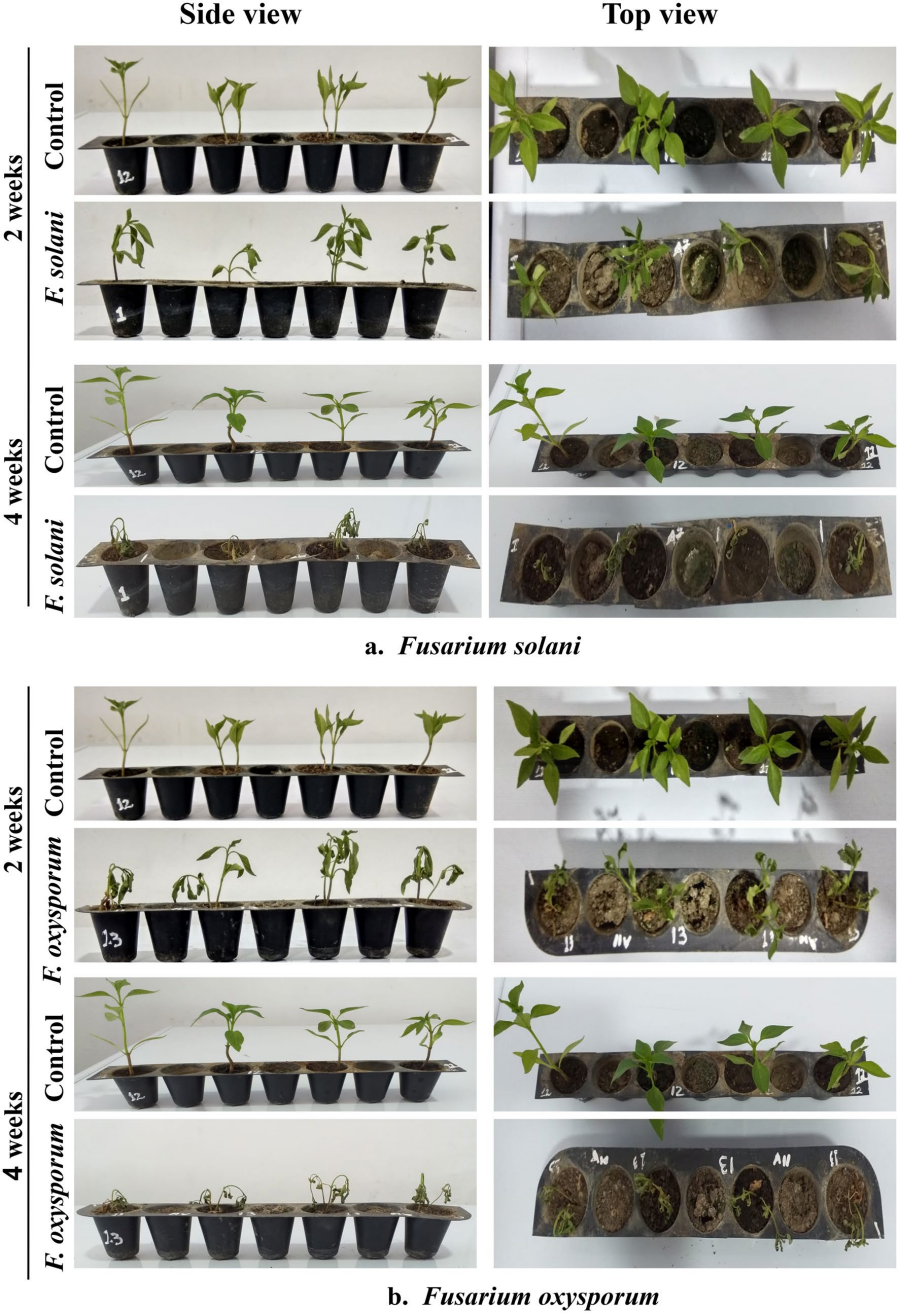
(**a**) Pathogenicity test of isolated *Fusarium solani* on potted chilli plants. (**b**) Pathogenicity test of isolated *Fusarium oxysporum* on potted chilli plants.

**Figure 6:**
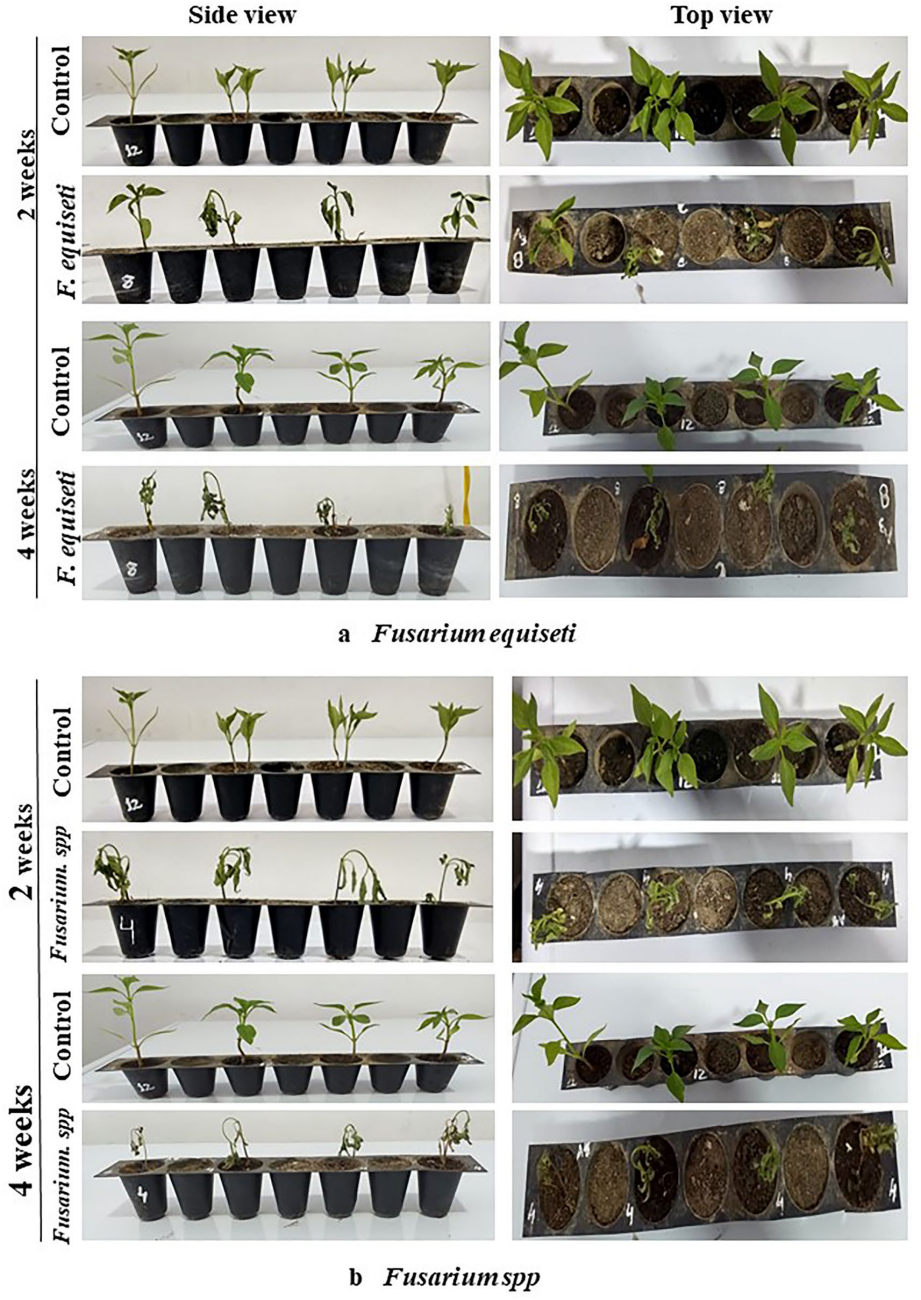
(**a**) Pathogenicity test of isolated *Fusarium equiseti* on potted chilli plants. (**b**) Pathogenicity test of isolated *Fusarium sp.* on potted chilli plants.

**Figure 7:**
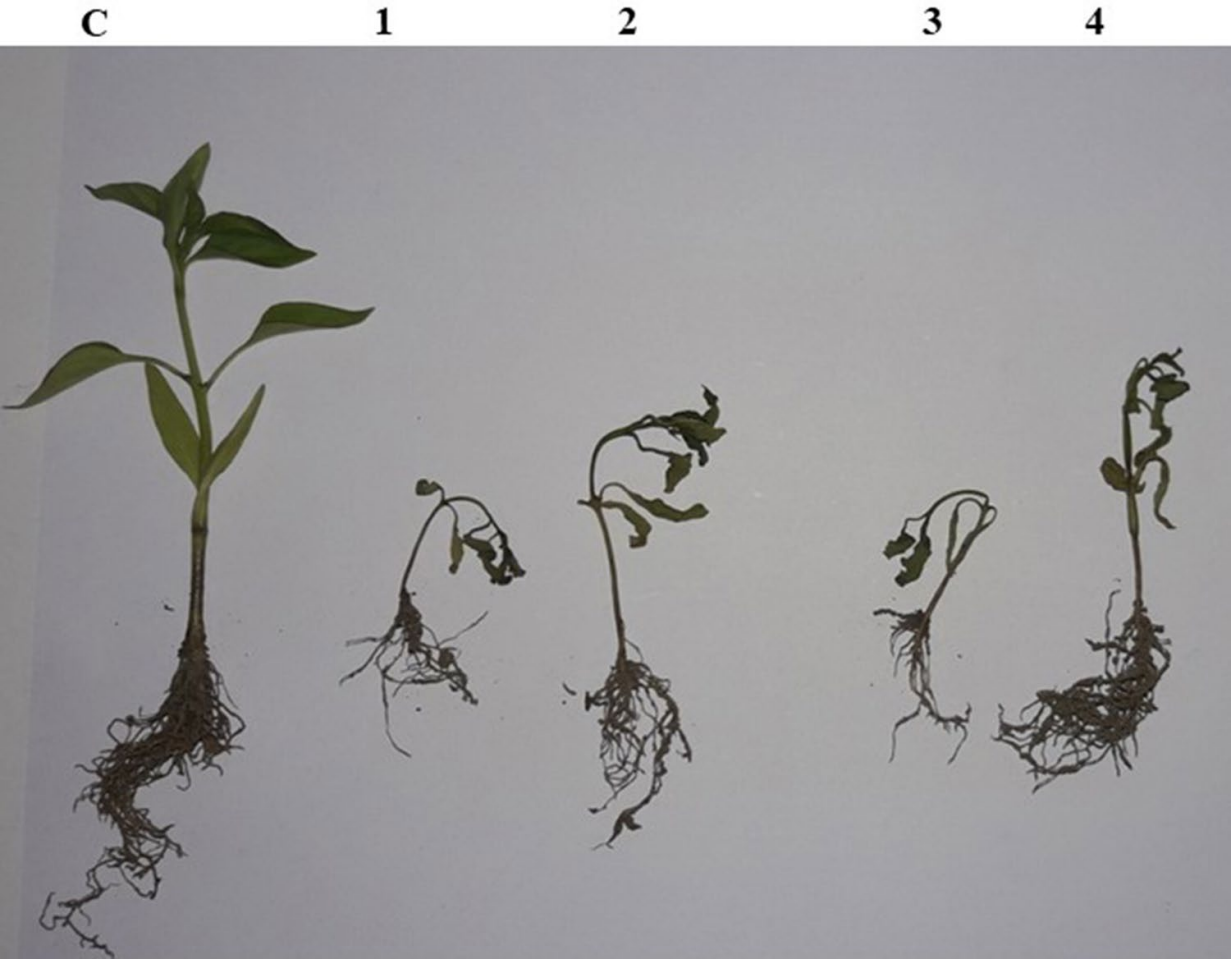
Symptomatological development of wilt diseases of chilli. C- control, 1*- F.equiseti,* 2*- Fusarium sp.,* 3*- F.oxysporum,* 4-*F.solani.*

**Figure 8:**
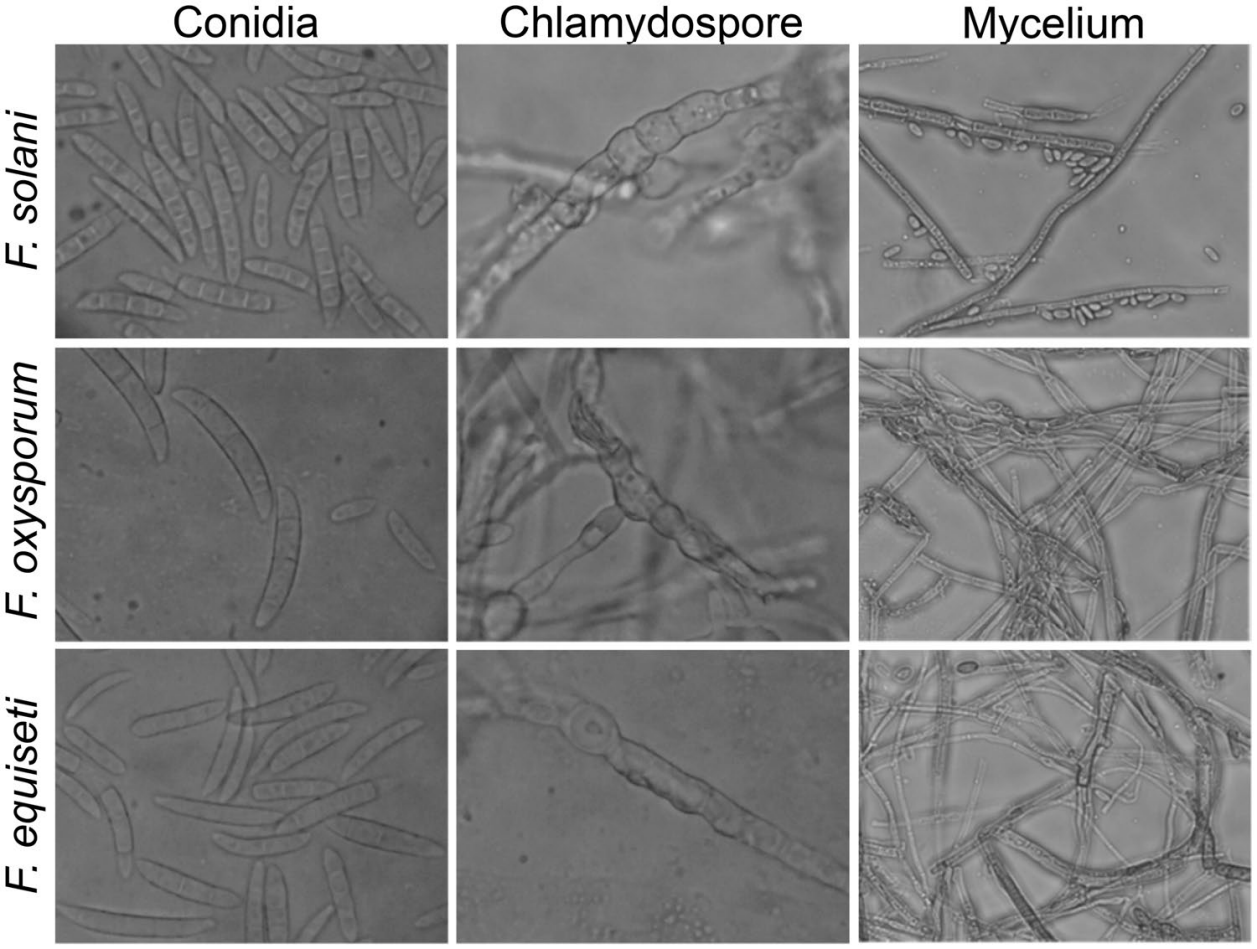
Comparative microscopic results depicting Conidia, Chlamydospore and Mycelium of the three fungi showing the difference.

The Pathogenicity of isolated fungi was established by confirming Koch’s postulates on potted chilli plants cv. ‘Shalimar Long’ (Figs. 5, 6). The initial disease symptoms in case of *Fusarium equiseti* inoculated seedlings developed after 10- 12 days of inoculation and in case of *Fusarium solani* and *Fusarium oxysporum* the initial symptoms were observed after 19–20 days of inoculation. The pathogens were re-isolated from the inoculated seedlings and compared with their original inoculated cultures. The re-isolated pathogens completely resembled the original inoculated fungi in their morphological, cultural and pathogenic characteristics, so satisfied Koch’s postulates. Pathogen inoculated plants developed initial symptoms as light green to yellowish discoloration of leaves (Fig. 7) followed by their shriveling, drooping and ultimately wilting leading to death of the whole plant. When the collar region of the plant was cut vertically, the vascular bundles showed brownish discoloration. Comparative microscopic results depicting Conidia, Chlamydospore and Mycelium of the three fungi show clear difference (Fig. 8).

### Molecular Characterization and phylogeny

**Figure 9:**
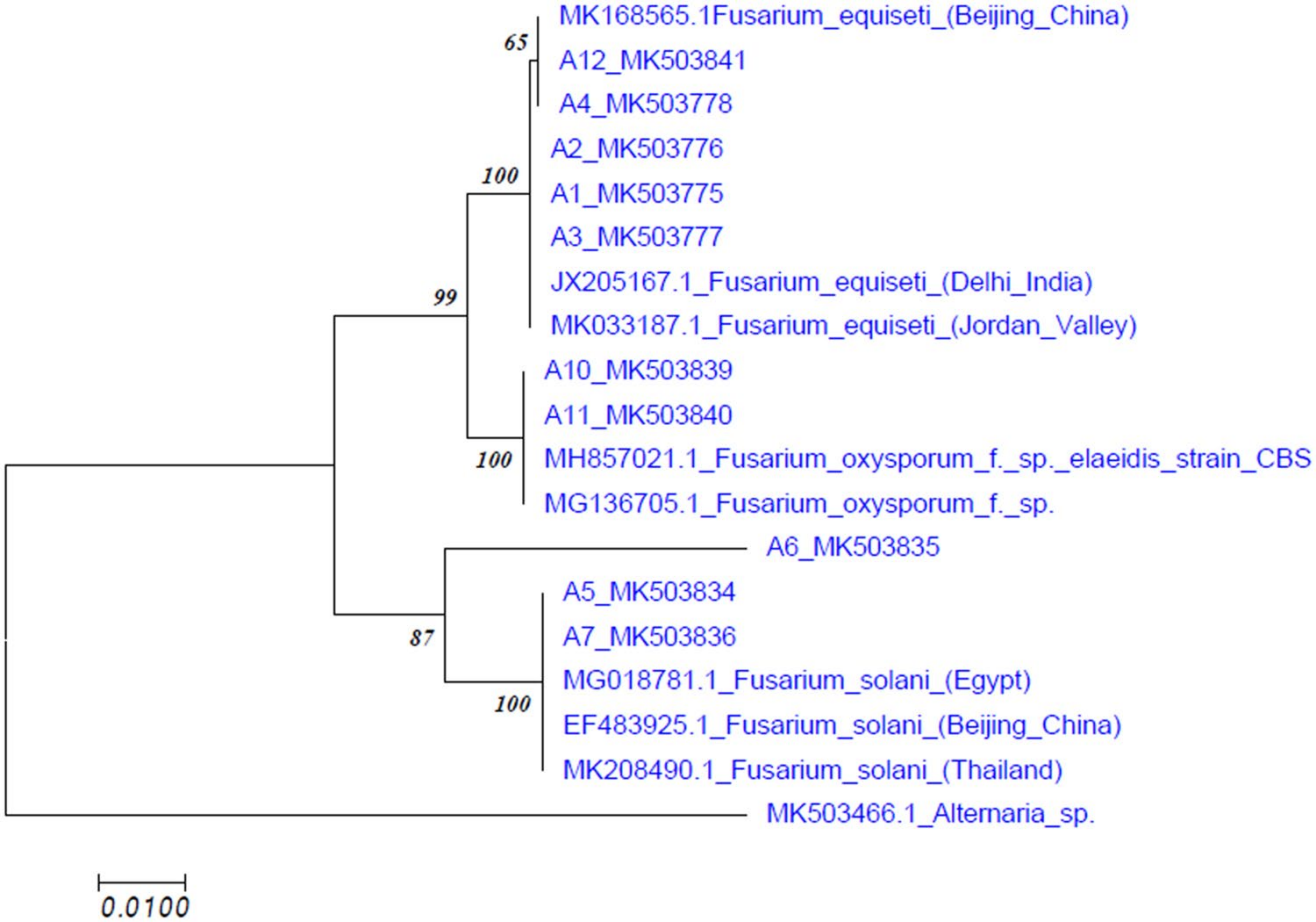
Dendrogram generated by ITS sequence data predicting similarity and evolutionary relationship between the isolates.

After sequencing the PCR product and analyzing with BLASTn, all sequences showed 98%-100% sequence homology with GenBank sequences. Sequences were submitted to NCBI GenBank and accession numbers were obtained (Table 1). Phylogenetic analysis revealed that our isolates clustered along with other submitted *Fusarium* isolates from GenBank. The sequences of *Fusarium* isolates formed same cluster with *Fusarium equiseti, Fusarium solani* and *Fusarium oxysporum,* but in separate subclusters. *Alternaria* formed a different cluster (outgroup) in the dendrogram (Fig. 9). We observed that using ITS region of the 10 isolates collected from different locations of J&K showed sequence homology with isolates reported from different regions particularly isolates of India, China, Egypt and Thailand.

## Discussion

Chilli is a widely grown crop all over the world for its flavor and colour; and is believed to have many health beneficial properties like anti-inflammatory and antioxidant potential^41^. However, from past few years the production of chilli has been hampered due to many prevailing abiotic and biotic stresses which include many bacterial and fungal diseases^42^. Among the fungal diseases most dominating is *Fusarium* species to which 50–80% losses in the production are attributed^43^. The pathogens causing wilt in chilli in the regions of Kashmir valley were isolated and later confirmed as *Fusarium oxysporum, Fusarium solani* and *Fusarium equiseti* based on the morpho**-**cultural characteristics as mentioned by Booth et al.^44^ as well as by sequencing results. The disease was prevalent in all the surveyed locations with varied levels of incidence which ranged from 22 to 55 per cent. The disease incidence was highest in district Budgam (52.10%) followed by Pulwama (48.70%) and Srinagar (35.20%). However, the incidence was least (26.70%) in district Anantnag. *Fusarium equiseti* has been reported to cause wilt in cauliflower in china and wilt in cumin reported from parts of India^45^ and has not been yet reported to cause chilli wilt in Himalayan region of Kashmir valley. On the basis of morpho-cultural characteristics, pathogenicity and comparison with authentic description given by Booth^44^, Domsch et al.^46^, Brayford^47^ and Zhu et al.^48^ the pathogens were identified as *Fusarium Oxysporum* Schlecht., *Fusarium equiseti* (Corda.) Sacc. and *Fusarium solani.*

All the three isolates viz., *F. oxysporum, F. solani, F. equiseti* isolates evaluated within the present study were infective in nature with slight variation in virulence. The appearance of the specific symptom/disease severity varied depending on the pathogenicity or virulence level of specific isolate of fungus. The fungal pathogens isolated from wilted chilli plants, when artificially inoculated through rhizosphere inoculation technique on potted chilli plants exhibited typical disease symptoms. Koch’s postulates were confirmed by re-isolating the pathogen from the artificially inoculated and infected plants. The plants developed initial symptoms on the second week of inoculation*.* The initial symptoms showed light green to yellowish discoloration of leaves followed by their shriveling, drooping and finally death of whole plant at fourth week of inoculation. The dried leaves remained clinged on the wilted plants. When the collar region of the plant was cut vertically, the vascular bundles showed brownish discoloration. The initial symptoms were first recorded in *F.equiseti* so, this strain of pathogen was more virulent as compared to other pathogens which were artificially inoculated (*F.oxysporum, F.solani*). *F*.*oxysporum* has also been reported from various regions of Kashmir valley and from many parts of India^49^ *F.solani* has been also reported from many parts of India^50,51^. *F. equiseti* was shown to be prevelent in five different locations in Kashmir valley.

In the present studies, the internal transcribed spacer (ITS) amplification with genera and/or species specific ITS primers, clearly differentiated all the three isolates of fungi into *F. oxysporum, F. solani, F. equiseti.* In a recent study PCR when carried out on *Fusarium* found in *Capsicum* species using ITS regions, the sequences successfully resulted in identification of five *Fusarium* species as *F. solani*, *F. oxysporum*, *F. equiseti*, *F. incarnatum*, *F. chlamydosporum* showing dominance in *F. solani* and *F. equiseti*^52^. Based on amplification and size of the amplicons in different isolates, amplified with ITS primer combinations viz., ITS1F and ITS4Rdirected the amplification of ~ 500 bp ITS**-**rRNA uniform amplicons in all the isolates of *F. oxysporum , F. solani, F. equiseti.* These ITS primer mixtures, which targeted and amplified a specific gene/region within the internal transcribed spacer (ITS) regions, are used for straight forward and early identification of fungal species viz., *F. oxysporum, F. solani, F. equiseti* from the infected plant tissues. The PCR products of ITS1-5.8S-ITS2 gene were sequenced and the pairwise and multiple alignment of pathogens identified through sequencing was carried out by Clustal Omega and later were published in Gene bank. (https://www.ebi.ac.uk/Tools/msa/clustalo/). While comparing the query sequence of ITS1 5.8S-ITS2 with the database sequence. Tree based method (Neighbour joining., NJ) were applied to test the efficacy of twelve DNA barcodes candidates for the identification of fungal species. Sequences were assembled, edited and multiple sequence alignments were performed using the ClustalW tool from MEGA 7.0 and a NJ tree, which was recommended as the standard barcoding method which was adopted and constructed using MEGA 7.0 software. In the present investigation three query sequences of different fungal species which were identified through sequencing were clustered together according to their similarity with their respective hits viz., *Fusarium equiseti*, *Fusarium solani*, *Fusarium solani* showing closeness with each other also.

## Conclusion

In the present study, three isolates of fungi were collected and isolated from diseased root/stem samples of chilli the pathogenicity was demonstrated on very vulnerable chilli plants. The pathogenicity of the contagious spp. isolates was confirmed based on the capacity of each isolate to cause ailment and appearance of the particular side effects viz., yellowing, wilting or plant death. Morphological characteristics of the causal pathogen were studied both on host as well as on artificial culture medium to identify the associated pathogen. Gene sequencing studies of ITS1-5.8S-ITS2 gene were elucidated and all the sequences were submitted in GenBank (NCBI). The major chilli growing hotspots of Kashmir Valley were investigated in the current study. Among many pathogens *F. oxysporum* and *F. solani* and *Fusarium equiseti* were found to be prevalent in these areas. *Fusarium equiseti* incidence is reported for first time in Kashmir valley.
