# Author Correction: Morpho-molecular identification and first report of *Fusarium equiseti* in causing chilli wilt from Kashmir (Northern Himalayas)

**DOI:** 10.1038/s41598-021-88867-4

**Published:** 2021-04-22

**Authors:** Ammarah Hami, Rovidha S. Rasool, Nisar A. Khan, Sheikh Mansoor, Mudasir A. Mir, Nazeer Ahmed, Khalid Z. Masoodi

**Affiliations:** 1 Transcriptomics Laboratory (K-Lab), Division of Plant Biotechnology, Sher-e-Kashmir University of Agricultural Sciences and Technology of Kashmir, Shalimar, Srinagar, Jammu and Kashmir 190025 India; 2 Division of Plant Pathology, Sher-e-Kashmir University of Agricultural Sciences and Technology of Kashmir, Shalimar, Srinagar, Jammu and Kashmir 190025 India

Correction to: *Scientific Reports* 10.1038/s41598-021-82854-5, published online 11 February 2021

The original version of this Article contained errors in Figures 3, 4 and 6.

In Figure 3, the labels for panels a and b were modified and “old fungal colony grown on PDA” was removed. The original panel c “Sporodochium” was incorrectly included and has now been removed. As a result, panels c, d and e were originally listed as panels d, e, and f.

In Figure 4, the labels for panels a and b were modified and “old fungal colony grown on PDA” was removed. The original panel c “Sporodochium” was incorrectly included and has now been removed. As a result, panels c and d were originally listed as panels d and e. Additionally, panel c “Microconidia” was incorrectly captured and is now rotated 90 degrees clockwise. Furthermore, panel e “Chlamydospore” was omitted from the original version of the Article.

Finally, in the label of Figure 6a,

“Fusarium equesiti”.

now reads:

“Fusarium equiseti”.

The original Figures 3, 4 and 6 and accompanying legends appear below. The original Article has been corrected.

**Figure 3:**
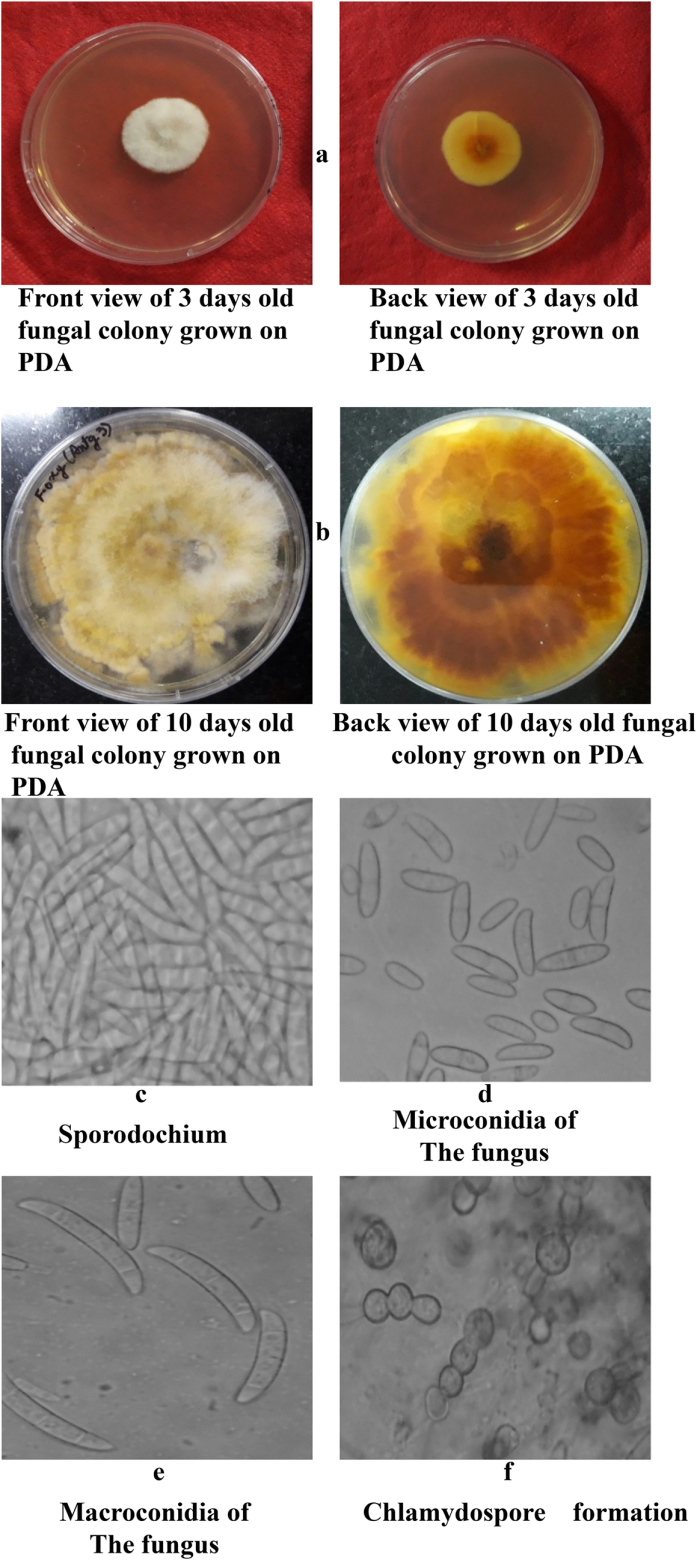
Morpho—cultural characteristics of *Fusarium oxysporum* Schlecht. Causing chilli wilt.

**Figure 4:**
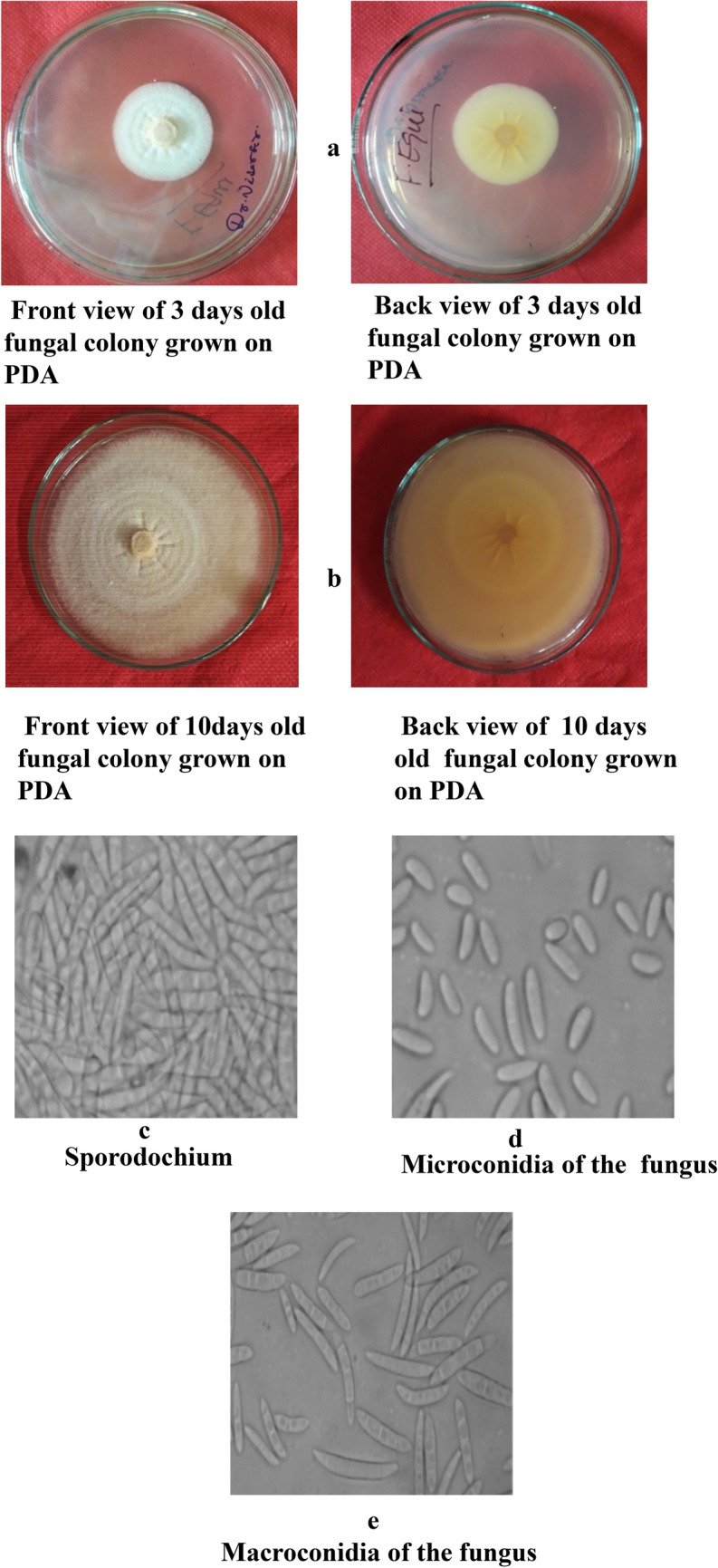
Morpho—cultural characteristics of *Fusarium equiseti* (Corda.) Causing chilli wilt.

**Figure 6:**
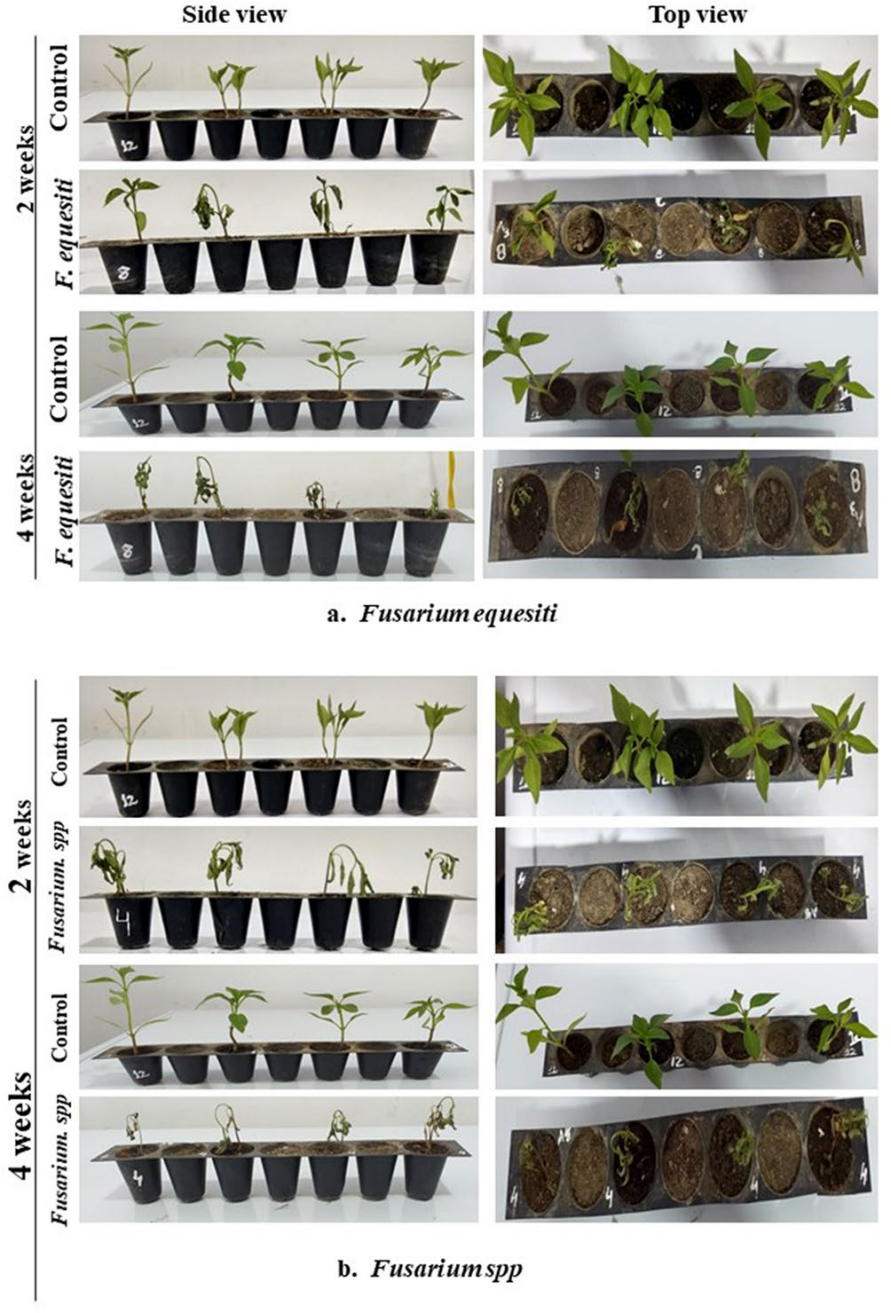
(**a**) Pathogenicity test of isolated *Fusarium equiseti* on potted chilli plants. (**b**) Pathogenicity test of isolated *Fusarium sp.* on potted chilli plants.

